# Partially-covered fractal induced turbulence on fins thermal dissipation

**DOI:** 10.1038/s41598-022-11764-x

**Published:** 2022-05-12

**Authors:** Soon Hong Chew, Su Min Hoi, Manh-Vu Tran, Ji Jinn Foo

**Affiliations:** 1 School of Engineering, Monash University Malaysia, 47500 Bandar Sunway, Malaysia; 2 Faculty of Engineering and Technology, Tunku Abdul Rahman University College, 53300 Kuala Lumpur, Malaysia

**Keywords:** Mechanical engineering, Fluid dynamics

## Abstract

The impacts of partially-covered fractal grids induced turbulence on the forced convective heat transfer across plate-fin heat sink at Reynolds number *Re*_*Dh*_ = 22.0 × 10^3^ were numerically and experimentally investigated. Results showed that partially covered grids rendered a higher thermal dissipation performance, with partially-covered square fractal grid (PCSFG) registering an outstanding increase of 43% in Nusselt number relative to the no grid configuration. The analyzation via an in-house developed single particle tracking velocimetry (SPTV) system displayed the findings of unique “Turbulence Annulus” formation, which provided a small degree of predictivity in the periodic annulus oscillations. Further assessments on PCSFG revealed the preferred inter-fin flow dynamics of (i) high flow velocity, (ii) strong turbulence intensity, (iii) vigorous flow fluctuations, (iv) small turbulence length scale, and (v) heightened decelerated flow events. These features stemmed from the coupling effects of multilength-scale fractal bar thicknesses in generating a veracity of eddy sizes, and a vertical segmentation producing heightened mass flow rate while inducing favourable wake-flow structures to penetrate inter-fin regions. Teeming effects of such energetic eddies within plate-fin array unveiled a powerful vortex shedding effect, with PCSFG achieving fluctuation frequency *f* = 18.5 Hz close to an optimal magnitude. The coaction of such traits limits the growth of fin boundary layers, providing superior thermal transfer capabilities which benefits the community in developing for higher efficiency heat transfer systems.

## Introduction

Turbulences are described as flows that possess irregular, unpredictable and chaotic fluid motions. The formation of turbulence links closely to the behaviour of particles, whereby excessive kinetic energy in portions of fluid are able to overcome the viscosity effects that dampens flow fluctuations^1^. It is encountered in everyday phenomenon and has great mixing capabilities due to intrinsic diffusive characteristics of increased rate of mass, momentum and energy transport. Such mixing properties heightened the flow-thermal boundary layers reconstruction and reshuffling probability, thus enhancing forced convection. To date, multitudinous approaches had been conducted to unravel the heat transfer oriented flow patterns. The usage of 2D planar space-filling grids have seen an increase in reputation for its effectiveness as a turbulator, owing to the feasibility of fine-tuning grid geometries in expressing for a preferred thermo-fluid interplay, especially the renowned fractal grid designs.

Fractals consist of self-similar geometrical structures that diminish in size, forming iterations of complex patterns^2,3^. Its dimension is defined using non-integral fractal dimensions *D*_*f*_, which are different with Euclidean geometry, whereby integer dimensions 0, 1, 2 and 3 are used to represent points, lines, surfaces and cubes, respectively^4^. Usually, fractal theories are utilized to describe objects that are irregular and disordered in nature, with the intentions to model roughness with finer details, such as describing for fibrous porous media^5,6^. Recently, fractal patterns had been widely integrated in heat transfer enhancement applications due to the increasing demand of high efficiency devices. For instance, simulated^7^ and experimental^8^ works demonstrated the use of fractal-tree-shaped fins in improving the performance of a latent heat storage (LHS) unit. It was shown that the energy discharge performance of LHS unit had been significantly augmented through the incorporation of fractal designs^7,8^. 2D planar fractal turbulators were also introduced for turbulence management, with the pioneering study conducted by Hurst and Vassilicos^9^. Their findings proposed the existence of a turbulence production and decay regions, which was later supported by Mazellier and Vassilicos, during their attempt to profile the centreline streamwise turbulence intensity as a function of wake-interaction length scale^10^. Owing to the promising nature of fractal grid induced fluid flow perturbation via the fine-adjustment of grid parameters, it is seen to be implemented in a variety of work, which includes but not limited to impinging jets^11–13^, flame speed augmentation^14^, and energy harvesting^15,16^.

Square fractal grids (SFG) in particular have been extensively researched due to its distinctive nature of turbulence production and decay regions^13,17^. It was reported that the turbulence generated from multilength-scale fractal grids attained higher turbulence intensities and local flow Reynold number *Re* with reference to a typical regular grid of similar or higher blockage ratio *σ*_*r*_^10,18^. Wakes shed by the grid bars of different length-scales meet at different downstream distances, and thus elongating the turbulence production region, enabling higher heat transfer capabilities. As such, Melina et al.^17^ conducted a thorough investigation on the forced convection of a cylindrical pin in the turbulence production and decay region, and was found that SFG obtains higher heat transfer performance under high *Re* conditions. Such auspicious results motivated the work by Hoi et al.^19,20^, whereby the team numerically investigated the correlation of fractal-induced turbulence on the forced convective heat transfer augmentation of plate-fin heat sink. It was reported that the placement of optimized SFG along the wind tunnel allows an increment in Nusselt number of plate-fin heat sink by 6.1% with respect to the reference^20^. The findings highlighted the special characteristics achieved by incorporating fractal designs, and a strong conjunction between the variables, namely turbulence intensity *I*, flow velocity *U*, turbulence length scale *L*_*t*_ and inter-fin spacing *δ* was determined.

However, the numerical results revealed that the hindrance from multitudinous wake interactions could retard the airflow velocity^20^. Such wake formation rises from the SFG largest grid bar, which introduce substantial flow recirculation that reduces turbulence intensities at inter-fin region. Hence, it raised the following questions: Are there any other fractal designs that could induce better turbulence structure that enhance heat dissipation performance? Will it be possible to maintain the advantages of SFG-induced turbulences, whilst improving heat sink thermal transfer, even at a targeted localized region? What will be the corresponding fluid flow structures induced through such newly designed grid? The outcome of these queries would propose a novel grid model that could provide additional insights in the preferred flow dynamics (i.e. effective turbulence management) for thermal dissipation. For the current investigation, it was hypothesized that a vertical segmentation to SFG may amass the production of highly positive thermal dissipation flow structures that are advantageous for the forced convective heat transfer of a plate-fin heat sink, paving the way for the development of higher efficiency heat transfer systems.

## Methods

**Figure 1:**
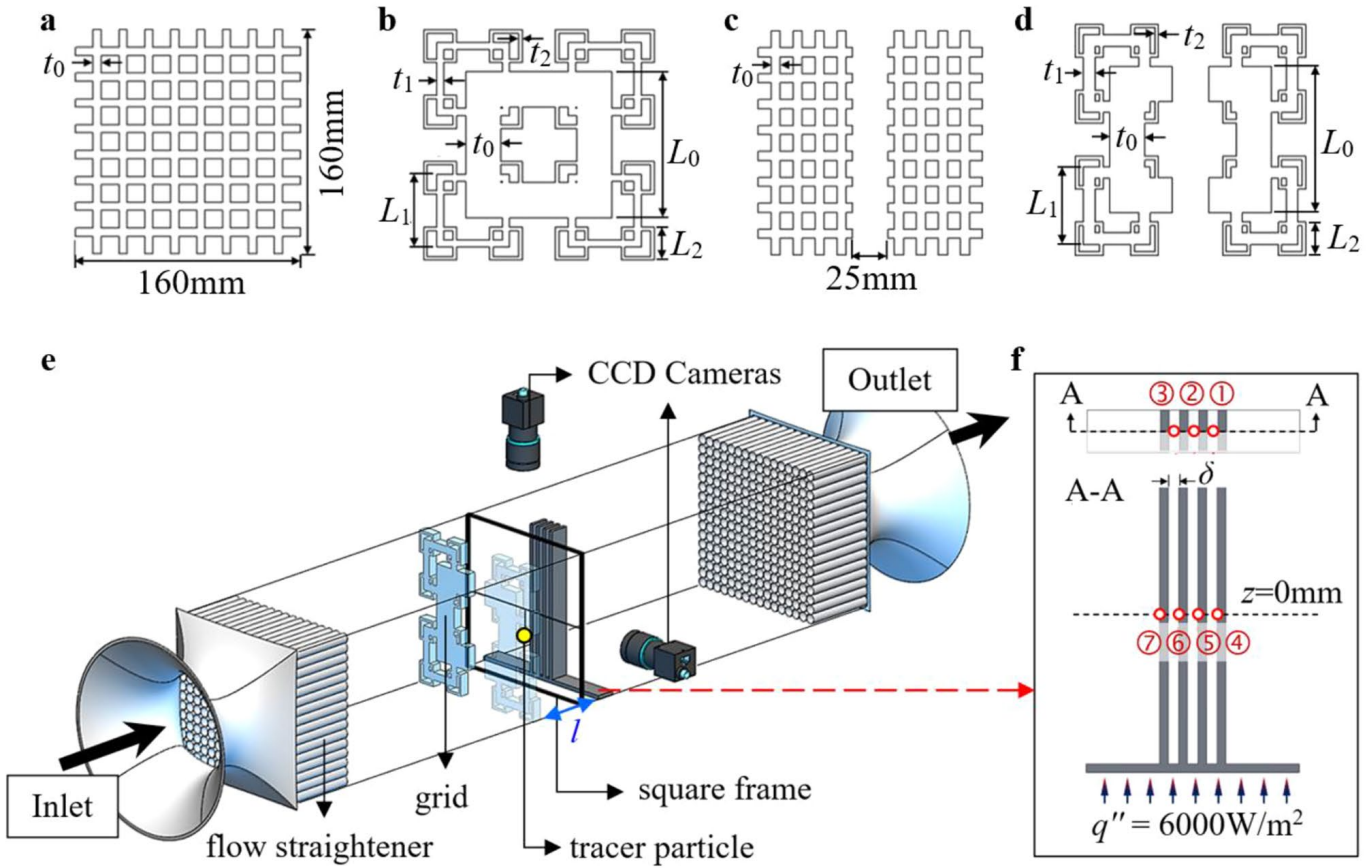
Schematic diagram of 2D planar space-filling gird of (**a**) RG, (**b**) SFG, (**c**) PCRG, (**d**) PCSFG, (**e**) geometrical representation of wind tunnel test section with tracer particle attached on square frame and (**f**) seven *T*-type thermocouple locations on the plate-fin heat sink.

Current investigation offers the means of acquiring an in-depth realization of positive thermal dissipative flow characteristics. As such, numerical and experimental approaches will be utilized to deduce and explain the grid-induced plate-fin heat transfer performances, as well as the underlining inter-fin flow dynamics. Briefly, a transparent acrylic wind tunnel of dimension 160 × 160 × 2560 mm^3^ was connected to a flow-straightened bell mouth inlet, with an axial fan (Kruger, SG) paired along to ensure centreline inlet air velocity *U*_0_ = 2 ms^-1^, corresponding to Reynold number of *Re*_*Dh*_ = 22.0 × 10^3^. The use of turbulator includes (a) regular grid (RG), (b) square fractal grid (SFG), (c) partially-covered regular grid (PCRG) and (d) partially-covered square fractal grid (PCSFG) as shown in Fig. 1a–d, with no grid (NG) configuration acting as the control. The fractal design follows simple recursive mathematical expression in defining for their physical dimensions in each fractal iterations *N*, and the SFG bears *D*_*f*_ = 1.86, as calculated using Eq. (1):^9^

1 $$D_{f} = \frac{\log B}{{\log \frac{1}{{R_{L} }}}}$$

where *B* signifies the number of patterns in iteration *N*, and *R*_*L*_ the length ratio *L*_*N−1*_/*L*_*0*_. All grids enjoy similar blockage ratio *σ*_*r*_ = 0.49 and details of the grids’ dimensions, i.e. thickness ratio *t*_*r*_ and fractal bar length *L* can be observed in Table 1. Each grid configuration together with a 1 × 4 rectangular plate-fin heat sink (aluminium 1100-H14) of dimension 4 × 20 × 160mm^3^ fabricated symmetrically to a comparable heated base plate were positioned within the wind tunnel test section as shown in Fig. 1e, with the inter-fin spacing *δ* and grid-fin distance *l* deduced at 5 mm and 10 mm, respectively. A heater plate (GUNT, DE) was utilized to provide constant base heat flux of *q''* = 6 × 10^3^ Wm^−2^. As such, the instantaneous temperatures at locations illustrated in Fig. 1f were measured using seven *T*-type thermocouples and recorded through a data logger (GL800, US) for a span of five minutes (steady-state). The average Nusselt number *Nu* of the plate-fin was then calculated with Eq. (4) to empirically evaluate the performance of forced convection, as shown below:

2 $$D_{h} = \frac{{4A_{w} }}{P}$$

3 $$\Delta T = T_{m} - \frac{{T_{in} + T_{out} }}{2}$$

4 $$Nu = \frac{{q^{^{\prime\prime}} D_{h} }}{{k_{air} \Delta T}}$$

where *D*_*h*_ denotes the hydraulic diameter, *A*_*w*_ the wind tunnel cross-sectional area, *P* the cross-sectional perimeter, *T*_*m*_ the mean temperature of plate-fin heat sink, *T*_in_ the inflow temperature, *T*_out_ the outflow temperature, and *k*_air_ the air thermal conductivity.

**Table 1:** Parameters of the grids in Fig. 1a–d.

Grid type	*t*_*r*_	*N* = 1		*N* = 2		*N* = 3	
		*L*_0_ (mm)	*t*_0_ (mm)	*L*_1_ (mm)	*t*_1_ (mm)	*L*_2_ (mm)	*t*_2_ (mm)
RG	1.00	160.00	5.76	-	-	-	-
PCRG	1.00	160.00	6.50	-	-	-	-
SFG	9.77	101.43	24.45	50.71	5.00	22.86	2.50
PCSFG	9.77	101.43	24.45	53.71	8.00	22.86	2.50

The setup was also modelled as a computational domain in numerical investigation using the computational fluid dynamics (CFD) commercial software package ANSYS-Fluent (ver.0.16.0, USA). Reynold Stress Model (RSM) was employed under the first order upwind spatial discretization to offer the prediction of grid-induced turbulence. The convergence criteria of residual 10^–3^ were prescribed for all the governing computational calculations, with the exception of energy equation to be 10^–6^. Details of the governing equations can be referenced in Teh et al.^21^. Even though RSM requires higher computational power as compared to most eddy-viscosity model, it carries an advantage of predicting anisotropic and inhomogeneous turbulence with higher degree of accuracy by solving additional transport equations of Reynolds stresses individually^22,23^. Of the important parameter includes the pressure-strain correlation term which principally describes the Reynold stresses energy production and transport processes, mean velocity gradient field interactions, and fluctuation of velocity field, which are crucial in elucidating the turbulent flow structures^23,24^.

Besides, mesh independency test using finer tetrahedron elements around the grid-fin conformation was also conducted, with maximum percentage difference of 0.48% recorded for *Nu* as the number of elements increased from 0.8 × 10^6^ to 3.0 × 10^6^. In all cases, reliable numerical outcomes were secured. Consequently, two main parameters, namely (*δ*, *l*), were explored for the design of experiment (DoE) to systematically observe the individual influence and correlation with respect to grid-fin’s *Nu*(*δ*, *l*), as the variables directly affect the compactness of a heat exchanger unit. Hence, a total of 55 sampling points were customary inputted (in a uniform manner) to the design space, and Kriging regression was applied to predict the response surface mapping with respect to CFD processed DoE data points. Kriging regression is a multidimensional interpolation acting upon with a polynomial model which is suitable for highly non-linear output response, such as turbulence affected characteristics^25,26^. As such method merely provides an approximation for the actual possible scenarios, 40 additional verification points were complemented into the design space to attest for the veracity of results. In particular, each verification point shall undergo similar CFD simulation, and the newly calculated result-set were collated with response surface whereby the percentage differences were determined. Through verification processes with all the different grid configurations, a maximum percentage discrepancy of 2.58% was warranted for the *Nu*(*δ*, *l*), demonstrating the accuracy of the current 3D surface mapping in describing for the theoretically simulated results.

Subsequently, a re-scaled transparent acrylic plate-fin setup (replacing aluminium heat sink) was employed together with an in-house developed single particle tracking velocimetry system (SPTV) to empirically capture grid-induced flow fluctuations. SPTV introduced the means of investigating localized flow structures in a non-intrusive and inexpensive approach. It captures the concatenating spatial position of a tracer particle that is lightly attached to a polyester yarn fluctuating at an inter-fin ‘local’ region with a pair of synchronized high-speed cameras (see Fig. 1f). The particle was imaged by two coupled charged devices (CCD) cameras (FLIR Integrated Imaging Solution Inc., CA) on top and side sections of wind tunnel at 80 fps, resulting in the compilation of 4.82 × 10^3^ images per camera. Image processing and correction methods were then performed using internally established MATLAB (R2016b, US) algorithm to acquire highly contrast particle images that were exempted from cameras’ distortion, refractive and perspective misplays. Such features allowed for the accurate detection of particle’s centroid in consecutive images through built in computational algorithm. Through comparison on the locality of tracer particle in successive images, the spatial positions can be reconstructed in a Cartesian coordinate system, where the particle’s trajectory, velocity fluctuations along with the underlining flow dynamics can be computed accurately. Details of the calibration and correction processes are shown in the following sections.

### SPTV multi-camera image processing

**Figure 2:**
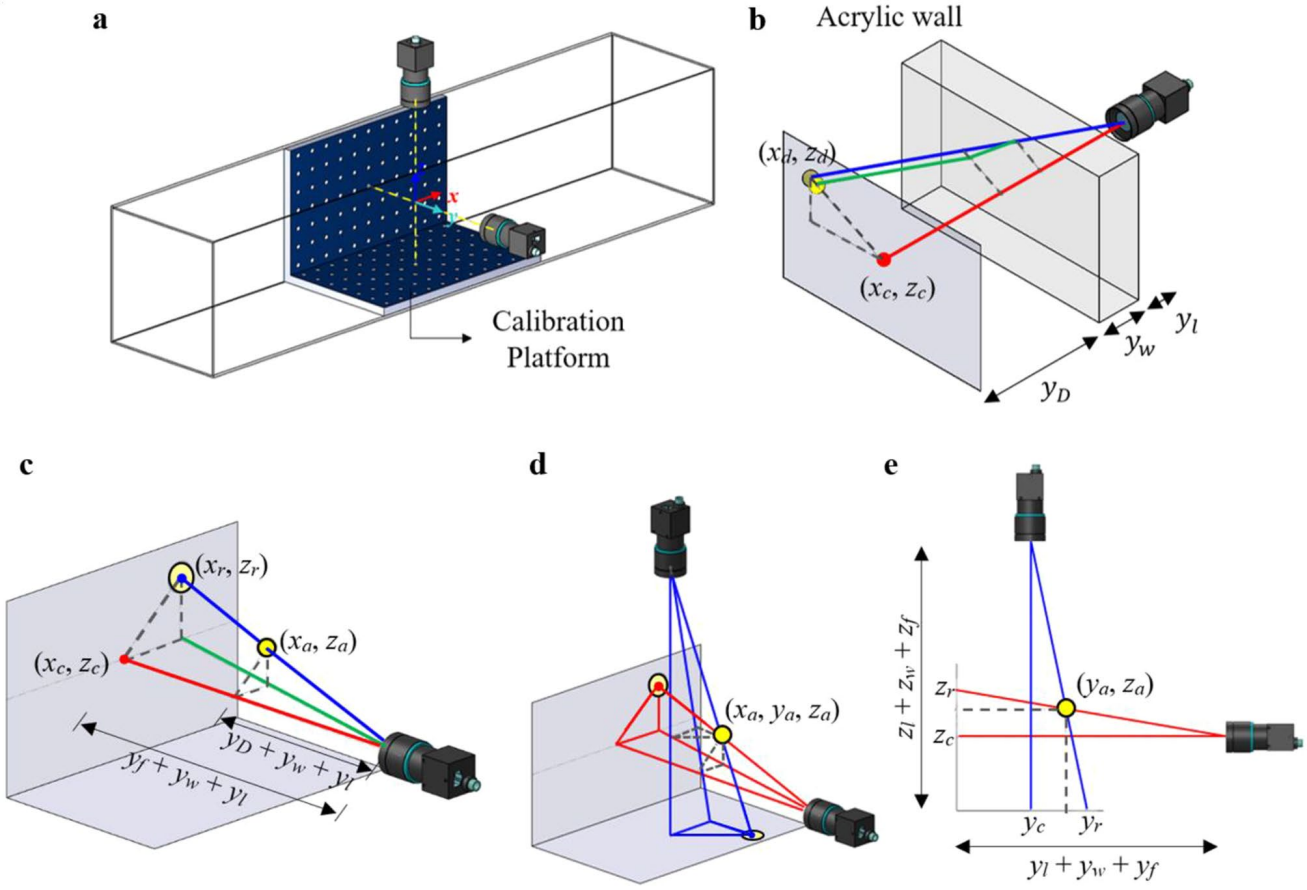
(**a**) Alignment of cameras using 7 × 11 circle array calibration platform, with the Cartesian coordinate set at intersection of cameras’ optical axis. (**b**, **c**) demonstrate the notation used for refractive and perspective error correction processes. Note that the perspective error influences the (**d**) projected coordinates of the two cameras, and (**e**) is the corresponding *Y*–*Z* view of the projection.

Two synchronized high-speed cameras were calibrated to ensure the precise alignment of optical axes at orthogonality with one another by incorporating a calibration platform (see Fig. 2a). The calibrator consisted of 77 white circles uniformly arranged at a 7 × 11 array in both *X–Y* and *X–Z* planes, where frontal images of the circles were captured and analysed using computational algorithm to attest for the maximum deviation of 0.10 mm between optical axis and platform centre, thus assuring parallelism of cameras’ optical plane with the platform. The intersection between the optical axes denotes the origin of the coordinate system, with (*x*, *y*, *z*) representing the streamwise, spanwise and transverse directions, respectively. Thereupon, the particle’s fluctuating positions were captured. The individual time-series images were further processed by an un-distortion algorithm in MATLAB single-camera calibration toolbox to minimize distortion effects. Briefly, images of checkerboard with square arrays of 5 × 8 were first recorded at 20 different orientations using the two cameras and registered subsequently in the toolbox. Consequently, the calibration parameters, i.e. focal length, optical centre and lens distortion coefficient were pinpointed, whereby a custom algorithm was realized to undistort all images, securing the SPTV particle’s spatial accuracy.

The aforementioned images were further processed to increase the contrast of the tracer particle through background subtraction and bitonal conversion techniques. Individual pixels of the images embodied a pixel value that varies between 0 (black) to 255 (white), with numerals in between representing monochrome colour tones. Each pixelized value was subtracted with the background (image with absence of tracer particle) to minimize background noises. Subsequently, an intensity threshold was introduced to binarize the images, i.e. setting pixel value above threshold to 1 (white) and vice versa, so that the contrast of tracer particle was emphasized, thus attaining a high signal-to-noise image for particle identification. The centroid of the particle was then determined by employing circular Hough transform, which is accessible through the MATLAB built-in function.

The centroid of tracer particle determined at current stage is subjected to refractive and projection errors. Concisely, refractive errors occur due to the bending of light as it passes between mediums of different optical densities. The detected particle positions will be observed to offset from the actual position, as illustrated in Fig. 2b. As such, a corrective module that utilizes Snell’s law had been incorporated into the MATLAB algorithm to tackle the inaccuracies [see Supplementary Section S1]. On the other hand, the perspective distortion caused different magnification of particle image in relative to the distance from lens (see Fig. 2c). Notably, the pixel-to-distance ratio varied at different planar locations, where particle situated closer to the lens would appear to be enlarged, and a small shift in particle coordinate would emerge as a large motion as detected by camera. Such repercussions were minimized by uncovering the perspective projection equations [see Supplementary Section S2]. Generally, all the time-series images of tracer particle were perceived to be projected onto a focusing frame coincident with the calibration platform. Since the dimensions of the calibration platform were discerned, the metric distance of the particle from optical centre can be calculated through pixel-to-length conversion. By locating the depth of particle in relative to the camera lens, the correct metric scale can be determined, hence an accurate spatial coordinate reconstruction can be computed. However, the depth information at current stage depends on the complementary action between both cameras, and would still be subjected to minor perspective inaccuracies. By observing on Fig. 2d, e, the particle’s coordinate in reference to both cameras can be related by means of similar triangles. Hence, an iterative method had been utilized to pinpoint the particle’s coordinate [see Supplementary Section S3]. The coordinates information from both cameras are allowed to iterate until a convergence criteria of *ε* = 1.0 × 10^–2^ mm is obtained. Combining information from both cameras, an accurate particle coordinate (*x*_*a*_, *y*_*a*_, *z*_*a*_) can finally be realized. As such, the instantaneous velocity fluctuation in the three different directional components can be computed, as defined by the change of displacement over a small time interval between frames. The employment of SPTV allows for the detection of turbulence characteristics, and are further extracted for analysis purposes.

## Results and discussions

### RSM and SPTV validation processes

**Figure 3:**
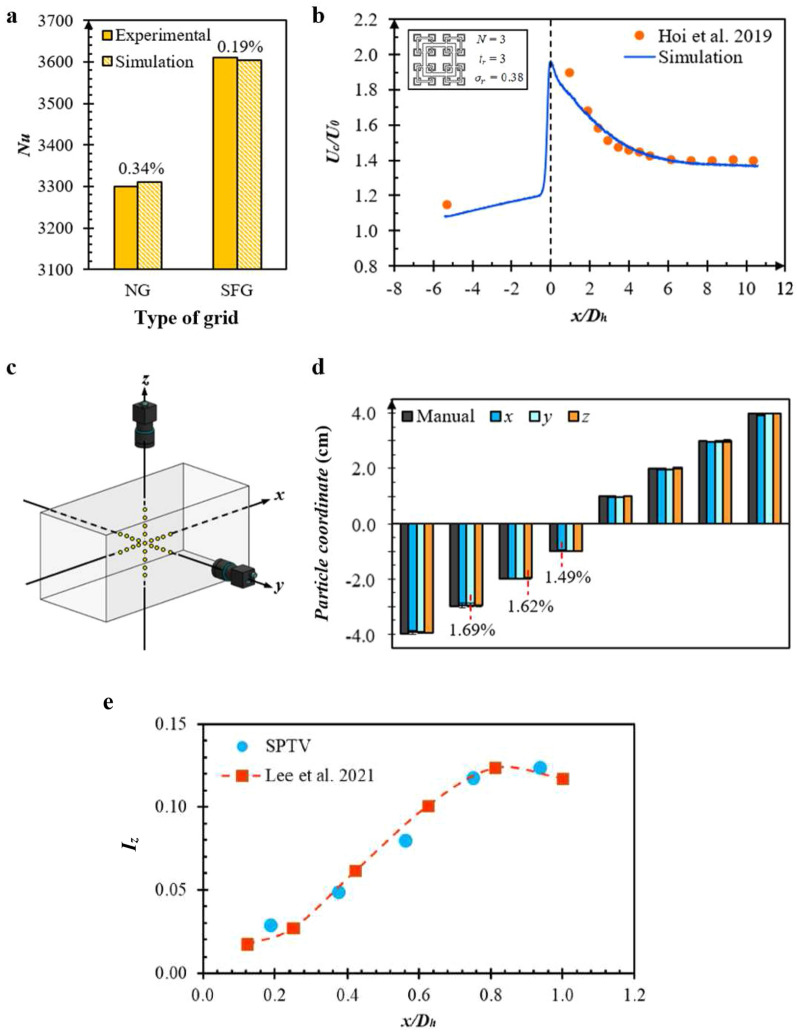
Top, numerical validation of (**a**) *Nu* for NG and SFG, as well as (**b**) the normalized centreline velocity of fractal grid generated turbulence against Hoi et al.^19,27^ experimental data. Middle, (**c**) geometrical representation of particle placements in the wind tunnel test section along with (**d**) the SPTV recorded error margins. Bottom, (**e**) SPTV validation of SFG induced *I*_*z*_ against Lee et al.^28^ experimental data.

In order to confirm the numerical accuracy of current RSM in revealing the fundamentals of insert-induced turbulence upon fins forced convection, careful validation using Hoi et al.’s^19,27^ experimentally recorded measurements are conveyed. Figure 3a demonstrates the comparison between the experimental study of NG and SFG induced *Nu* with present numerical simulation. Evidently, low percentage differences of 0.34% and 0.19% are recorded for NG and SFG, respectively. Likewise, fractal grid simulated flow dynamic, namely, the centreline streamwise flow velocities *U*_*c*_ at different *x/D*_*h*_ are as well validated and compared with experimental data. It is seen in Fig. 3b that the normalized flow velocity at the lee of grid decreases × 0.28 nonlinearly, i.e., from about *U*_*c*_*/U*_*0*_ = 1.9 to 1.4, with a maximum discrepancy of 6.15% recorded between the numerical and experimental results. The modest difference corresponds to magnitude of *U*_*c*_*/U*_*0*_ = 0.08, which is believed to be affected by the coarsening of mesh size at distances afar from grid at *x/D*_*h*_ = −5.3 to ensure timely computation. But nevertheless, such subtle documented differences in Fig. 3a, b indicate that the RSM simulated forced convection and fluid flow fits well with the experimental study. Hence, it is justified that the current use of numerical scheme is capable of predicting grid-induced flow dynamic in aiding the thermal dissipation of plate-fin heat sink with reasonable accuracy.

Similarly, validation on the accuracy of the current SPTV coordinate detection and reconstruction system is also conducted. The SPTV tracer particle had been manually complemented into the wind tunnel test section at known spatial positions. Figure 3c demonstrates the careful arrangement of tracer particle along the (*x*, *y*, *z*) axes, with each placement bearing a step size of 1 cm, expanse from the origin. Subsequently, particle images are recorded at each coordinate and analysed using MATLAB algorithm, whereby the error margins can be computed (see Fig. 3d). Clearly, the maximum percentage errors attained for each directional component are discerned to be 1.49%, 1.69%, and 1.62%, respectively, which corresponds to the highest recorded inaccuracy of 5.0 × 10^−2^ cm. In addition, the SPTV particle detected transverse turbulence intensity *I*_*z*_ is validated against the experimental results shown in the work of Lee et al.^28^ (see Fig. 3e), whereby the SFG induced centreline turbulence characteristics were detected by means of piezoelectric thin-film flapping velocimetry approach. Clearly, the current SPTV detected *I*_*z*_ fairly agrees with the reported experimental data, with the maximum deviation recorded to be 8.24 × 10^–3^. Such minute misplays imply a high accuracy and reliability in spatial reconstruction and turbulence detection, thus validating the accuracy of current SPTV characterizing grid-induced turbulences.

### Effects of *δ* and *l* on fins forced convection

**Figure 4:**
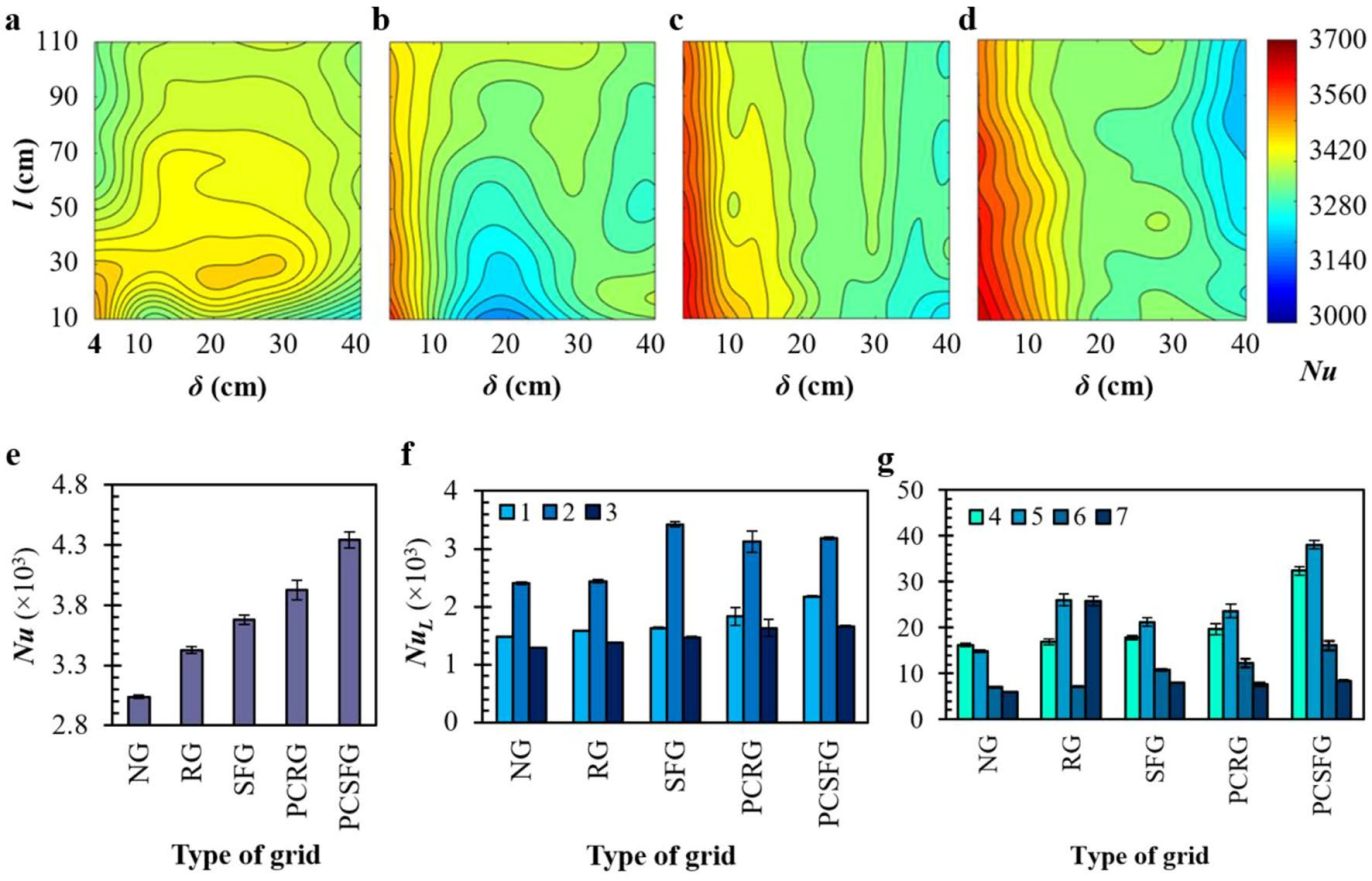
Top, the numerical 2D *Nu* contour plot induced by (**a**) RG, (**b**) PCRG, (**c**) SFG and (**d**) PCSFG as a function of *l* against *δ*. Bottom, various grid empirically induced (**e**) *Nu*, along with the *Nu*_*L*_ of (**f**) base section and (**g**) mid-fin section of plate-fin heat sink.

From the numerical study, the correlations between *δ* and *l* upon plate-fin heat sink forced convection at *Re*_*Dh*_ = 22.0 × 10^3^ are acquired. As seen in Fig. 4a, the RG induced *Nu* expresses its unique forced convection as a function of *Nu*(*δ*, *l*) with a wider high *Nu* coverage amongst all grid configurations that spans between 4.0 mm ≤ *δ* ≤ 35.5 mm and 10 mm ≤ *l* ≤ 75.5 mm. RG achieved a rather consistent *Nu* distribution, owing to the uniform *t*_0_ assignment that generates wakes of highly homogenous and isotropic turbulence accompanied with lower flow dynamic disturbance. Such weaker wake interplays lessen the disruption to flow boundary layer along each fin, hence justifying the all-around lower capability in promoting thermal dissipation. On another note, SFG is observed to attain an extensive high *Nu* coverage in comparison with RG, but smaller in the expanse of partially-covered grids (see Fig. 4b). Such enhancement in *Nu* contrasting to RG is likely to be contributed via the multi-length scale interactions of wakes that originated from SFG’s variety fractal bar thicknesses. Clearly, increasing the parameters (*δ*, *l*) is observed to have deteriorating effects in *Nu*, indicating that the thermal dissipation performance is highly sensitive to the tuning of *δ* and *l*. Moreover, a weak *Nu* regime is determined at the span of 15 mm ≤ *δ* ≤ 25 mm and 10 mm ≤ *l* ≤ 14 mm. Essentially, small *δ* are capable of breaking down flow recirculation introduced by wake of largest length-scale fractal bar. The plate-fin physically disrupts SFG induced wake structures into smaller ones to allow for effective thermo-fluid interactions^20^. As such, turbulence structures will be able to attach more efficiently along the fins’ surface and support the advancement of thermal transfer. However, lengthening of *δ ≈* 20 mm broadly reduces this effect, mainly due to the deteriorating performance in breaking down large flow recirculation, subsequently affecting vital turbulence parameter such as weaker turbulence intensity and lower flow velocity between fins.

Interestingly, PCRG and PCSFG are able to realize an effective domain of high thermal dissipation, with higher *Nu*(*δ*, *l*) discerned around 4 mm ≤ *δ* ≤ 10 mm for the former and 4 mm ≤ *δ* ≤ 12 mm the latter (see Fig. 4c, d). The integration of larger *t*_*0*_-induced wakes (see Table 1) accompanied with fluid flow acceleration through the vertically aligned separation generates beneficial hydrodynamic interactions which gives rise to intense forced convection. Notably, the *Nu* is less sensitive towards *l*. The partially-covered grids induced flow perturbation brings about the highest local acceleration immediately leeward from grid. The lengthening of *l* increases the jet mixing in terms of spanwise and streamwise flow dynamic dissipation and diffusion which eventually retard slightly the fins’ thermal transfer. Yet, *Nu* decreases with increasing *δ*. The penetration of flow fluctuations into fins’ flow boundary layer may have been gradually weaken at larger *δ*, which supports unwanted fluid bypass. Surprisingly, PCSFG is able to mitigate the shortcoming of SFG, where the lowest *Nu* generated is observed at a region of larger *δ* and *l* (blue). This is made possible due to the ability of vertical segmentation in preventing the formation of sizeable flow recirculation. Absence of such recirculation accompanied by higher flow velocity reinforces the favourable multitudinous wake interactions initiated from fractal bars of different length scale, effectively empowering thermal dissipation performance. In general, the 2D *Nu* contours suggest that the implementation of partially-covered grids are capable of providing higher thermal dissipation even at a wider *δ*. The findings would imply the possibility of utilizing lesser fins through realization of larger *δ* during fabrication of heat exchanger unit with the implementation of partially-covered grid, whereby the material and manufacturing costs could be significantly cut down. To further assess such outcomes, the optimum *δ* = 5 mm and *l* = 10 mm are empirically evaluated in the next section for a deeper insight on the various grid-induced flow dynamics.

### Grids induced turbulence on fins forced convection

Undoubtedly, the experimentally proven highest *Nu* = 4341.7 is achieved using PCSFG as shown in Fig. 4e, with a remarkable percentage enhancement of 42.9%, as compared with the control NG. The augmentation in *Nu* is followed by PCRG, SFG, and RG ranked in descending order, with the percentage increase recorded as 29.2%, 21.0% and 12.8%, respectively. The attainment of *Nu* induced from different turbulators are comparable to the numerical results performed through RSM, thus uncovering the value of simulated studies in predicting for the trend of forced convective heat transfer on plate-fin heat sink through various 2D planar grids. Clearly, the current uses of partially-covered grids are superior to their classic grid counterparts in terms of heat transfer, suggesting the implementation of a vertical mid-plane separation incites favourable flow dynamics that support forced convection.

On another note, the localized Nusselt number *Nu*_*L*_ at locations mentioned in Fig. 1f are illustrated in Fig. 4f, g to identify the locations with the utmost heat transfer. Interestingly, the *Nu*_*L*_ displayed at the base of plate-fin for all grid configurations showed symmetricity, whereby the *Nu*_*L*_ surges at the base of mid fin (position 2). The highest achieved *Nu*_*L*_(2) is recorded for SFG owing to the presence of thick *t*_0_ assignment, which generates wakes of sizeable length-scale near the base region for vigorous thermo-fluid interplay. Yet, the effects slowly diminish as we shift the position to centreline, whereby PCSFG is now observed in Fig. 4g to dominate the forced convection through realization of potent *Nu*_*L*_ at inter-fin regions (positions 4, 5 and 6). In spite of the high attainment of *Nu*_*L*_, a steep decline is observed near the outer-fin surface (position 7) for SFG, PCRG and PCSFG. Current phenomenon might indicate the diverging of airflow afar from fins’ exterior, owing to the growth of flow boundary layer at leading edge of outer-fins. However, a dissimilar phenomenon is observed for RG, where *Nu*_*L*_(7) is noticed to be comparable with *Nu*_*L*_(5) but demonstrate a deterioration in *Nu*_*L*_(6). The evenly separated RG perforations with uniform *t*_0_ generated an alternating regions of high and low airflow velocity *U* lee of grid, producing an imprint of *t*_0_ in dissociating for retardation of flow. Such imprint is projected streamwise towards the fin, with position 6 likely to be coincident with it. The low flow kinetic energy reduces the likelihood of flow reshuffling, thus sabotaging the plate-fin local thermal dissipation. On another note, positions (5, 7) are integrated at the immediate region behind grid perforations, which surges the *Nu*_*L*_ due to the heightened flow acceleration. Nevertheless, the forced convection is noticeably greater at the mid of fins, i.e. positions (2, 5) for all grid conformations. Therefore, the SPTV tracer particle is implemented at centreline *x/D*_*h*_ = 0.125 to investigate the flow dynamics that dominates thermal transfer processes.

### Empirical flow dynamics at localized inter-fin region

**Figure 5:**
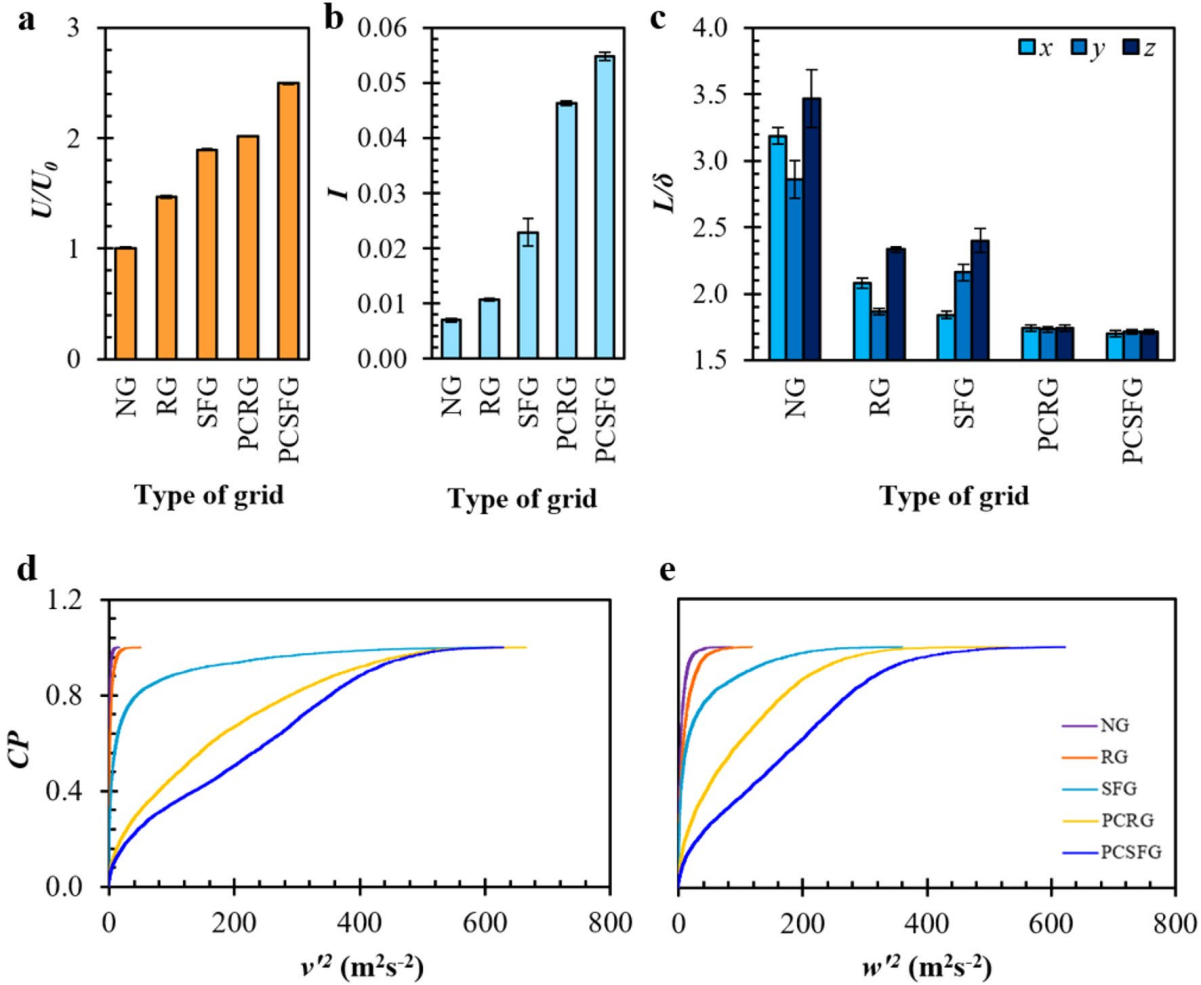
Various grid induced (**a**) *U/U*_0_, (**b**) *I*, (**c**) normalized *L*/*δ* in (*x*, *y*, *z*) directional components at centreline *x*/*D*_*h*_ = 0.125 and cumulative probability distributions of (**d**) *v*^’2^ and (**e**) *w*^’2^.

Figure 5a illustrates the *U* normalized with *U*_0_, i.e. *U/U*_0_ induced through the different 2D planar grids. The *U* is gauged by means of a hotwired anemometer (Testo 405i, DE) at centreline *x*/*D*_*h*_ = 0.125 in the absence of plate-fin array. Notably, the *U/U*_0_ generated through various grids exhibit similar trend as the *Nu*, with *U*(PCSFG) > *U*(PCRG), and *U*(SFG) > *U*(RG). It is renowned that the implementation of turbulator allows the acceleration of air flow due to the sudden contraction of flow passage. The introduction of a vertically aligned separation in the partially-covered grids promotes mid-plane jet formations, owing to the principle of mass conservation. Such separation effectively reduces the undesirable fluid bypass around the plate-fins, and forcefully regulates the working fluid to penetrate inter-fin regions. In addition, the accelerated airflow in between the fin array enforces greater wall shear stress along fin surfaces, which limits the growth of viscous sub-layer, thus effectively enhancing forced convection.

Even though the mid-plane separation is employed on both PCRG and PCSFG, a greater heat dissipation is still documented for the latter. The enhancement in *Nu* for PCSFG contrasting with PCRG may be due to the multi-length scale wake interactions via the multiplicity of fractal bar thicknesses. As reported earlier^17,29^, the interplay between accelerated airflow and wake structures through the variety of fractal bar thickness promotes generation of multi-length scale turbulent eddies. Small-scale eddies induced through thinner fractal bars are described to effectively facilitate plate-fin heat dissipation through flow dynamic energy cascading. The formation of anisotropic and inhomogeneous eddy structures encompassing a collection of sizes and frequencies increase the likelihood to the disruption of flow boundary layers along the fins. On the contrary, PCRG engenders weaker flow agitation capabilities due to the isotropic and homogenous turbulence formation by uniform *t*_0_ assignment. With the current employment of PCSFG, critical fragments of turbulent eddies may be filtered along the inter-fin regions, coupling with the high *U/U*_0_ triggers the active reshuffling of flow boundary layers, thus expounding the event where *Nu*(PCSFG) > *Nu*(PCRG).

Besides, 2D-planar turbulators generate wakes with intensified shear layers that lead to the formation of turbulence eddies. In order to quantify the regional turbulence intensity *I*, the root mean square of velocity fluctuations detected from SPTV is rationalized with *U*_0_:

5 $$I = \frac{{\sqrt {\frac{1}{3}\left( {u^{^{\prime}2} + v^{^{\prime}2} + w^{^{\prime}2} } \right)} }}{{U_{0} }}$$

where 〈*u'*^2^〉, 〈*v'*^2^〉 and 〈*w'*^2^〉 represent the ensemble average of squared velocity fluctuations in (*x*, *y*, *z*) directions, respectively. As illustrated in Fig. 5b, the paradigm demonstrated by *I* for the various grids followed closely to that of *Nu*, implying a positive correlation in between the two, i.e. higher *I* give rise to greater *Nu*. Evidently, the *I* induced from partially-covered grids are comparably more intense than that of the fully-covered grids, thereafter the control NG. As mentioned in the previous sections, raised level of *I* documented for PCSFG and PCRG are due to the elimination of flow recirculation due to the absence of grid bars at the centreline region. The highly accelerated air flow interacts directly with the plate-fin, restructuring favourable wake dimensions that provide intense energy necessary for vigorous turbulent eddies to infiltrate inter-fin regions.

**Figure 6:**
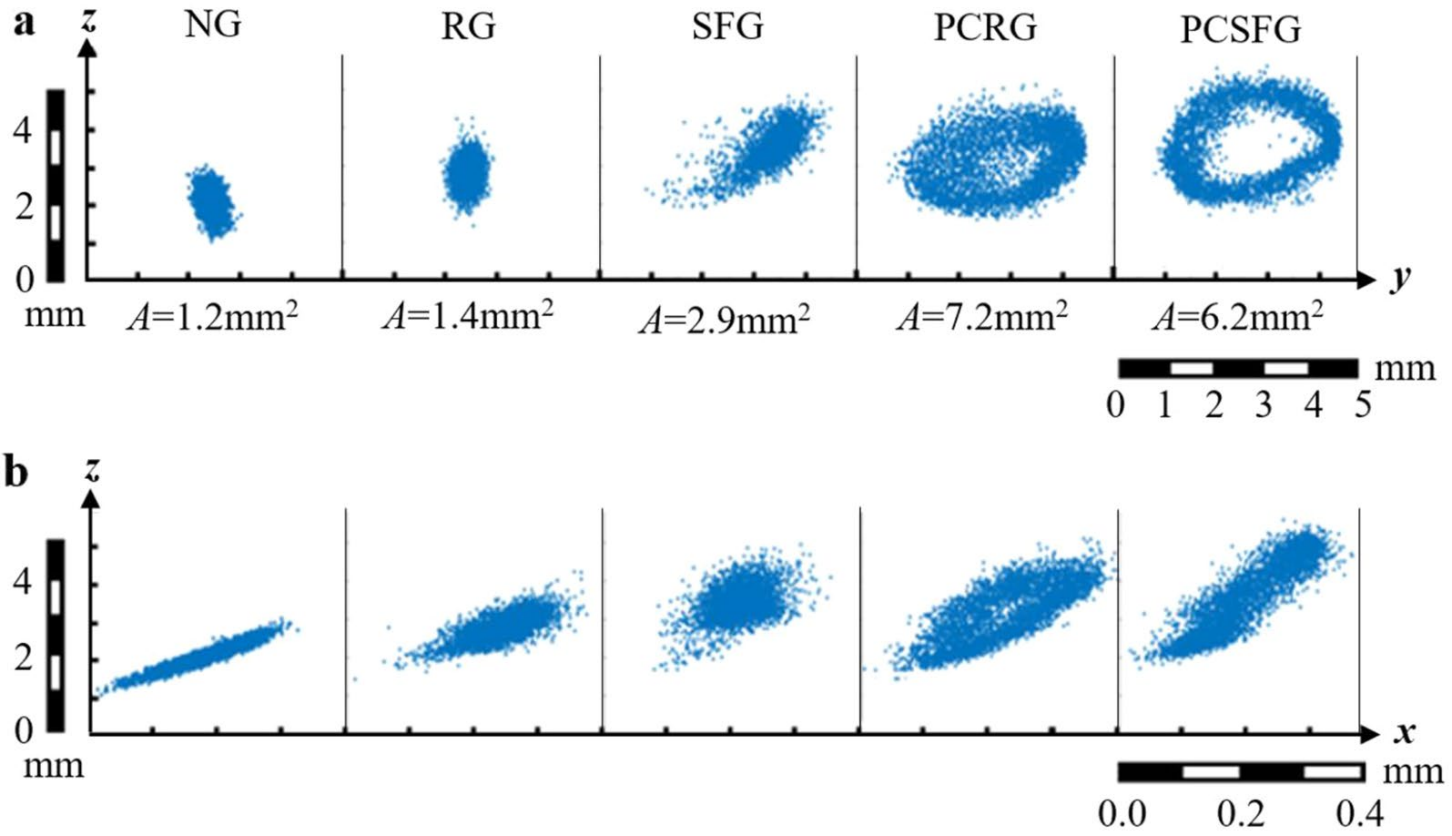
Trajectory of SPTV tracer particle in the (**a**) cross-sectional and (**b**) side view induced from various grid turbulence.

In order to conceptualize the effects of *I* induced from various grids, the trajectories of the tracer particles are demonstrated in Fig. 6a, b, with the former representing the *Y–Z* plane (cross-sectional view) and latter representing the *X–Z* plane (side view). Clearly, smaller fluctuations are observed for NG and RG configurations, whereby the frontal coverage areas *A* with respect to *Y–Z* plane are recorded at a minimum of 1.2mm^2^ and 1.4mm^2^, respectively. Through the amalgamation of fractal bars, SFG is observed to attain a greater *A* = 2.9 mm^2^ that fairly resembles the shape of a crescent. Interestingly, both partially-covered grids develops an elliptical structure from the particle’s trajectory, which would be fitting to address it as a ‘Turbulent Annulus’. The formation of the annulus may signify the generation of new and unique flow structures, as it displays small degree of predictability in the particle’s periodic annulus oscillations, despite the chaotic nature of turbulence. Nevertheless, higher fluctuation amplitude is observed from PCRG and PCSFG generated particle trajectory, especially in the *y*- and *z*- directions, which are vital in disturbing the flow and thermal boundary layers. The incompressible grid structures on two sides of turbulator generates multidirectional vortical eddies that provides substantial vibrational energy as indicated by annulus formation. Such powerful eddies are forcefully filtered within inter-fin regions, which effectively limits the growth of thermal boundary layers, thus increasing the probability of heat transfer through forced convection. Remarkably, the *A* of PCRG is greater than PCSFG despite the lower *I* attainment. Distinct flow dynamics induced from PCSFG consolidated the particle’s trajectory to a dense and intense flow fluctuation around the sizable annulus. The phenomenon may imply the multilength-scale eddies shredded from PCSFG are able to distribute the immense energy around the annulus circumference. As it fluctuates within inter-fin region, the energetic turbulence structures are able to adhere to the fins’ surface more effective, thus restructuring the flow boundary layers in a more precise manner. Unlike for PCRG, which has a smaller annulus dimension along with diffusive characteristic at the interior regime. Such diffusive effect disperses flow energy to undesirable intermediate regions and weakens the energy distribution around the annulus, further reduces the likelihood of boundary layer disruption, thus retarding heat transfer processes.

To further describe for the fluctuation intensities, cumulative probability (CP) distribution on the *v’*^2^ and *w’*^2^ for the variety of turbulators are depicted in Fig. 5d, e. As shown in Fig. 5d, NG and RG have a small range of spanwise fluctuations, whereby 95% of *v’*^2^ less than 3.8 m^2^s^−2^ and 10.7 m^2^s^−2^ are recorded, respectively. It showed a vast difference with SFG as 95% of *v’*^2^ is below 233.8 m^2^s^−2^, implying a great elevation in fluctuation amplitude. However, the strength is still inferior to the partially-covered grids, with PCSFG recorded with a higher probability of stronger fluctuations approximately between 80.0 m^2^s^−2^ < *v’*^2^ < 440.0 m^2^s^−2^ as compared to PCRG. Likewise in Fig. 5e, the overall *w’*^2^ trend depicted minor differences with *v’*^2^, where a clear distinction of wider *w’*^*2*^ variations are still observed for PCSFG, along with PCRG, SFG, RG and NG ranked in descending order. The event is apparent at the top five percentile, as PCSFG realized the highest variation of *w’*^2^ > 381.1 m^2^s^−2^. The pronounced *v’* and *w’* give rise to greater development of *A*, suggesting a larger area of flow boundary is being agitated at the localized inter-fin region, thus poses a unique advantage in supporting potent thermal dissipation. In addition, Fig. 5d, e showed that the fractal designs (SFG and PCSFG) generally produces greater variety of flow fluctuation as compared to the regular designs (RG and PCRG). The finding is crucial as it further supports the concept of PCSFG induced flows are capable to inherit turbulence features similar to that of SFG, whereby multilength-scale eddies are generated from the multitude fractal bar thicknesses and filtered within the fins. With the amalgamation of such feature and the added benefit of accelerated centreline airflow, PCSFG would induce highly effective turbulence structures in enhancing forced convective heat transfer, which is exceptionally suitable for localize cooling applications.

In the case of turbulent airflow, the effects of turbulence integral length scale *L* on the forced convective heat transfer should be considered as well, as it unveils critical thermo-fluid interplay upon heat exchanger thermal transfer^17,29^. *L* provides a representation of spatial dimension for turbulent vortices. Such *L* are computed by integrating the normalized autocorrelation function of velocity fluctuation with respect to the time-lapse, using Eq. (7):

6 $$R_{{f^{^{\prime}} }} \left( t \right) = \frac{{\left\langle {f^{^{\prime}} \left( t \right)f^{^{\prime}} \left( {t + \tau } \right)} \right\rangle }}{{\left\langle {f^{^{\prime}} \left( t \right)^{2} } \right\rangle }}$$

7 $$L = U_{0} \mathop \smallint \limits_{0}^{T} R_{{f^{^{\prime}} }} \left( t \right)d\tau$$

where *R*_*f’*_ represents the normalized autocorrelation function of velocity fluctuation, *f’* the directional components of velocity fluctuations (*u’*, *v’*, *w’*), *τ* the time-lapse, *T* the first zero-crossing of *R*_*f’*_*,* and < . > the ensemble average. The *L* generated from the different 2D-planar grids are rationalized with *δ*, i.e. *L*/*δ* and is shown in Fig. 5c.

Unsurprisingly, the NG induced *L*/*δ* in (*x*, *y*, *z*) directions are realized to be the highest. The straightened airflow is only subjected to wall shear stresses from wind tunnel surfaces, which lacks flow obstructions prior to plate-fin filtration, hence higher *L*/*δ*. Conversely, the *L*/*δ* is substantially reduced with the use of space-filling grids: a direct consequence of grid bars in filling the planar spaces. Interestingly, RG and SFG developed a comparable *L*(*z*)/*δ*, but are contrasting in reference to streamwise and spanwise. The event suggests that a sizeable *L*(*y*)/*δ* by SFG is preferred for forced convection since *Nu*(SFG) > *Nu*(RG), and could be enforced through the realization of smaller *L*(*x*)/*δ*. By considering vortices rotating in the *X–Z* plane, the dwindling *L*(*x*)/*δ* increases the vortices’ angular velocity around *y*-direction *ω*_*y*_, but in turns extending the vortex line laterally due to the conservation of angular momentum, i.e. vortex stretching. The elongation of vortex structures thus effectively interacts and disrupts the fins’ boundary layer. Similar findings were reported in Hoi et al.^27^, and it warrants the benefits of smaller *L*(*x*)/*δ* in enhancing heat transfer, as it encourages larger *L*(*y*)/*δ* formation to interrupt fins’ flow boundary layer more effectively. The implication concurred closely with RG induced *L*, yet poses an opposite effect of streamwise elongation, which directs the flow energy to undesired intermediate regime that lessened boundary layers’ agitation probabilities.

Nevertheless, an overall small *L*/*δ* is still favourable for forced convective heat transfer, since partially-covered grids are noticed to induce a relatively small and uniform *L*/*δ*. The findings are supported with reference to previous literatures^20,30–32^, whereby higher heat transfers are usually achieved at lower *L*. The contraction of flow due to the two partially-covered grid segments positioned along each side of wind tunnel could have developed a profound amount of similar yet counter-rotating pair of vortices. The vortex pair that oppose in motion maximize the straining of airflow, which induces highly intense eddies with smaller diameters. Such development of eddies could possibly be sorted along the fin surfaces, and promotes heat transfer through flow dynamic energy cascading.

In general, the coupling effects of (*U*_*high*_*, I*_*high*_*, L*_*low*_) as demonstrated from PCSFG induces flow structures that are favourable in enhancing heat transfer. The results expressed in the current section showed that SPTV is viable to provide informative data to describe for the preferred thermal dissipation flow dynamics. However, one might wonder if the investigation on a targeted localized region using SPTV particle at centreline of inter-fin is sufficient to conclude for the whole plate-fin array. Hence, numerical predictions are utilized in the following to describe for the overall flow dynamics at inter-fin in terms of isosurfaces and 2D contours to append for the limitations of current experimental findings.

### Simulated flow properties at inter-fin region

**Figure 7:**
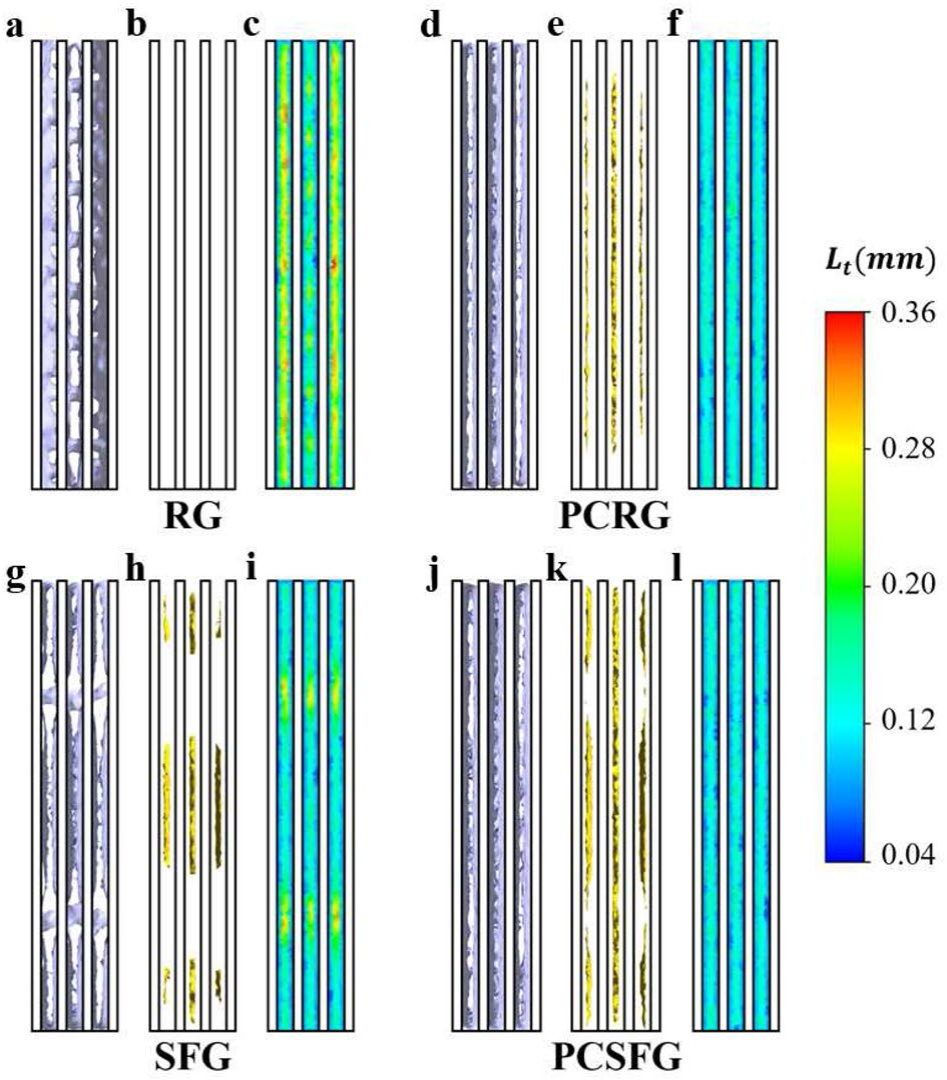
The cross-sectional isosurface representation of (**a**, **d**, **g**, **j**) *U/U*_0_ = 2.38, (**b**, **e**, **h**, **k**) *I* = 0.33; and (**c**, **f**, **i**, **l**) the 2D contour plot of *L*_*t*_ induced from various grid at *x*/*D*_*h*_ = 0.125. Note that *δ* = 5 mm and *l*/*D*_*h*_ = 0.0625.

The isosurfaces of (i) *U/U*_0_ = 2.38, (ii) *I* = 0.33 and (iii) 2D contour of turbulence length scale *L*_*t*_ at *x/D*_*h*_ = 0.125 are demonstrated in Fig. 7 to predict the underlying flow dynamics at a more pronounced inter-fin region. By focusing on RG in Fig. 7a, it is noticed that the high *U/U*_0_ is unlikely to penetrate through the plate-fin array. The evenly distributed perforations of RG generated low flow acceleration that is rapidly dissipated and dispersed with the surrounding tardy fluid momentum leeward of grid, disabling high *U/U*_0_ to be regulated into the inter-fin separations. Besides, the isosurface distribution of strong *I* in Fig. 7b is perceived to be partitioned into segments, with bigger voids arising between the second and third fins, owing to the *t*_0_ imprints from windward grid geometry. The findings supported the RG induced *Nu*_*L*_ as mentioned in Fig. 4g, whereby alteration of high and low *Nu*_*L*_(4, 5, 6) coincides agreeably well with alterations of the *I* isosurfaces, further supporting the positive correlation between *I* and *Nu*.

Moving on to SFG, it is noticed that the 3D isosurface of *U/U*_0_ in Fig. 7d is able to penetrate well through the inter-fin separations. The incorporation of airflow that forced through SFG of thicker *t*_0_ in conjunction with the unevenly distributed fractal dimension significantly stimulates the jet flows, producing working fluid of high flow velocity to predominantly infiltrate through the fin-array. Such flow structure increases the likelihood of flow perturbations, which contributed to the isosurface distribution of strong *I* as shown in Fig. 7e. Interestingly, the isosurface distributions for both *U/U*_0_ and *I* are perceived to inhibit high isosurface porosity, with voids scattered towards the upper and bottom portions of each inter-fin. The flow recirculated in the lee of fractal bars incurs considerable flow retardation, yielding an imprint of *t*_0_ dissociated isosurface. The amalgamation of such transition between the potent and frail interfaces may lessen the possible reshuffling of flow and thermal boundary layers along fins, which sabotage the local thermal dissipation.

Conversely, highly uniform *U/U*_0_ isosurface scattering for the PCRG and PCSFG can be distinctly seen in Fig. 7g, j, respectively. Apprehensively, jets are not only created and accelerated through the insert perforations via the sudden introduction of an incompressible grid, most importantly, the flow dynamics are as well simultaneously amassed around the mid-plane segmentation, which allows the air to speed up and penetrate deeper and more uniformly, hence increasing the scalar and flow momentum transportations. Moreover, denser isosurface distribution density can be clearly perceived with PCSFG as to PCRG. Notably, minor discontinuities are recorded for the former in comparison with SFG, suggesting the effect of multitudinous wake interactions in lowering down the flow velocity has been significantly mitigated. The strong *I* distributions of PCRG and PCSFG are of greater extent with seemingly the least variation in terms of respective uniformity (see Fig. 7h, k). Through effective coupling between the grid-induced turbulence and downstream plate-fin array, it initiates the second stage of flow eddies filtration, which leads to a predominant, unique fluid flow fluctuation along with potent flow dynamics to penetrate into fin-array. As a result, PCRG and PCSFG empower vigorous fin-wake interplay which effectively heighten forced convection.

Furthermore, the numerically calculated 2D *L*_*t*_ contours generated by the various grid conformations are investigated as well. Such numerically computed *L*_*t*_ is defined as^33^:

8 $$L_{t} = \frac{{C_{\mu }^{3/4} \kappa^{3/2} }}{\varepsilon }$$

where *κ* denotes the turbulence kinetic energy, *ε* the turbulence eddy dissipation rate, and $C_{\mu }$ a model constant of *C*_*μ*_ = 0.09. From Fig. 7c, f, RG configuration is clearly seen to incite the largest inter-fin *L*_*t*_, whereas domain of non-uniformity can be observed with SFG. Admittedly, the region of high *L*_*t*_ of both grids are associated with the corresponding *t*_0_ of the windward grid geometry. The sizable span of *t*_0_ gives rise to wakes of greater length scale, which generates substantial shear levels and subsequently bigger eddies via wake-flow hydrodynamic interplays. Conversely, SFG’s smaller scale fractal bars promote the production of smaller eddies, that may be potently filtered and disseminated to the *t*_0_ imprint complement regions. Interestingly, the small-scale eddies are determined in Fig. 7i, l to propagate and uniformly distributed for PCRG and PCSFG, similar to preceding findings in Fig. 5c. The absence of first iterative fractal bar in the mid-plane segmentation eradicates the accumulation of high length scale wakes, thus preventing the formation of large turbulent eddies directly windward of fins. Most importantly, miniscule yet vital fragments of turbulent eddies are found to approach and attached actively along fin surfaces, which may effectively facilitate plate-fin heat transfer through flow energy cascading, hence enhancing the forced convection process. In general, the amalgamations of (*U*_high_*, I*_high_*, L*_*t-*low_) are determined to be the favourable characteristics for potent thermal transfer processes. Such numerical findings coincide closely with empirically detected flow dynamics at centreline *x*/*D*_*h*_ = 0.125, justifying the current use of SPTV in describing for the positive thermal dissipation flow properties. Hence, statistical analysis is conducted for a rigorous analyzation on grid induced flow characteristics.

### Statistical analysis of turbulent flow

**Figure 8:**
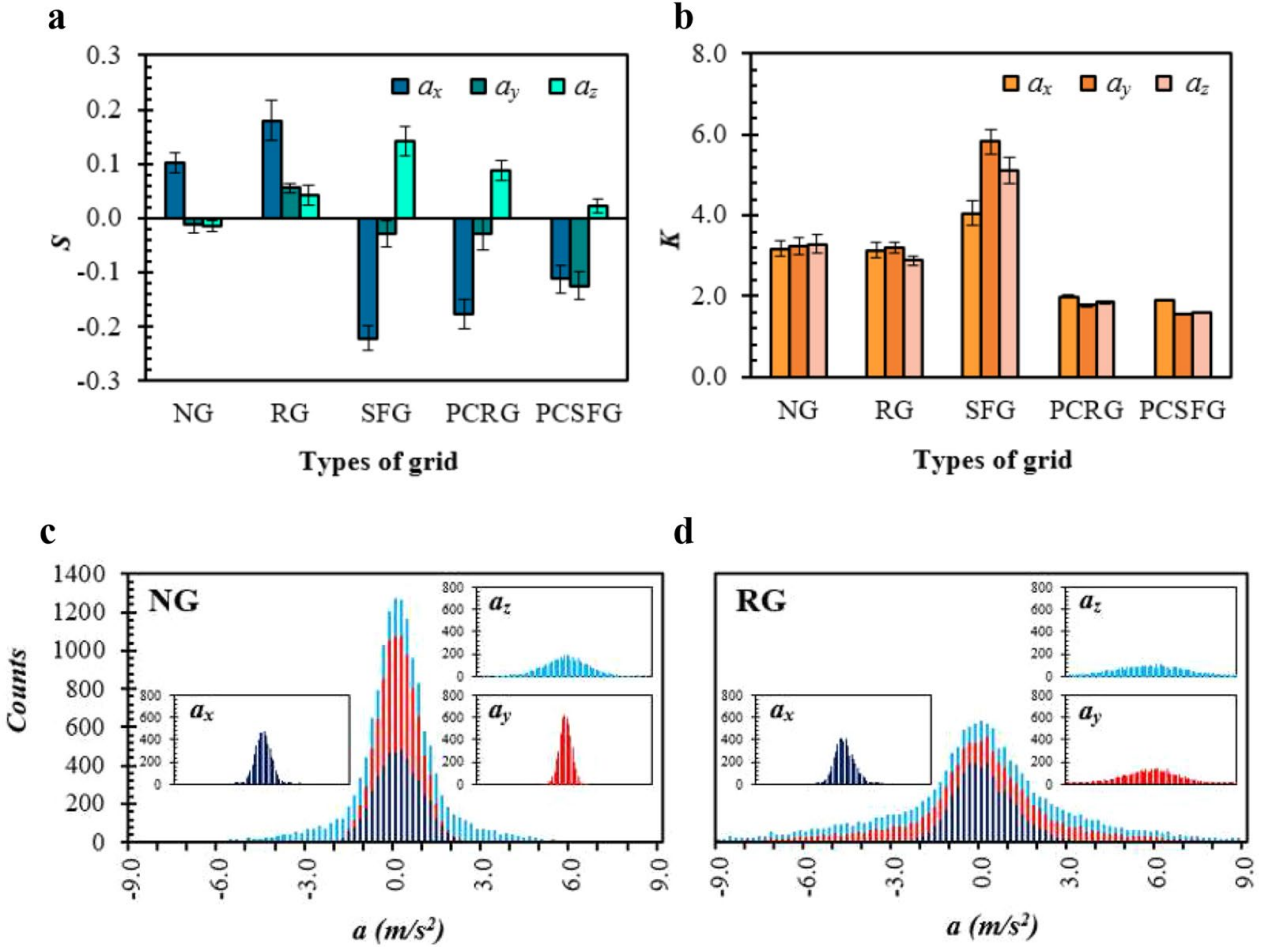
Top, various grid induced turbulence on (**a**) *S* and (**b**) *K* of SPTV particle acceleration in the (*x*, *y*, *z*) directional components. Bottom, the stacked histogram of acceleration components induced by (**c**) NG and (**d**) RG.

Skewness *S* and kurtosis *K*, which represent the symmetricity and the extremities of data distribution, are utilized to statically analyse the acceleration components induced from the planar grids (see Fig. 8a, b). It is computed in accordance with the equations:

9 $$S = \frac{{\left\langle {a_i^{~3}} \right\rangle }}{{\left\langle {a{{_i^{~2}}}} \right\rangle }^{\frac{3}{2}}}$$

10 $$K = \frac{{\left\langle {a_i^4} \right\rangle }}{{\left\langle {a_i^{2}} \right\rangle }^{2}}$$

where, *a* represents acceleration and *i* denotes the (*x*, *y*, *z*) directional components.

Undeniably, Gaussian distribution of data bears skewness and kurtosis values of 0 and 3, respectively. It is noticed in Fig. 8a, b that the acceleration distributions for NG and RG approximate to the suggested value, and the stacked histogram (see Fig. 8c, d) verifies that the acceleration components are rather normally distributed. The collected data follows closely to the characteristics of Gaussian distributions, whereby approximately (68.2 ± 1.2)%, (95.5 ± 0.7)% and (99.7 ± 0.2)% of all acceleration components induced from NG and RG falls within ± 1, ± 2 and ± 3 standard deviation from the mean, respectively. However, such components are slightly positively skewed, where *S*(NG) < *S*(RG). The results signify an increase in accelerated flow for RG as compared to NG, especially in the streamwise direction. Clearly, a non-Gaussian behaviour is realized for SFG, PCRG and PCSFG, whereby (|*S|*> 0, *K* > 3) are recorded for the former, and (|*S|*> 0, *K* < 3) for the latter duo. The ± *S* achieved by SFG implies extreme decelerated turbulent events documented in the (*x*, *y*) directions, along with turbulent accelerations in *z-*direction. These accelerations are considered to be rare and intensive, as indicated with the high positive *K* > 3. Interestingly, similar *S* developments are recorded for the partially-covered grids, but vastly disparate in *K* as evident from Fig. 8b. The realization of *K* ≈ 2 for PCRG and PCSFG depicts an increase in likelihood for the extreme decelerated (*a*_*x*_, *a*_*y*_) turbulent events, which could very well imply the high occurrences of alternating flow directions that escalates to the formation of copious vortices.

Undoubtedly, PCSFG is seen to manifest the highest negative skewness of *S* = −0.12 for *a*_*y*_, which may suggest the preference for exhibiting extreme decelerated flow structures in the lateral direction for a strong thermo-fluid interplay between highly potent and numerous collaborative vortices. The comparable negative *S*(*a*_*x*_, *a*_*y*_) indicated the small *L* eddies are compacted with intense *X–Y* plane flow circulations. Such crowding effects of dense and powerful eddies accompanied with minor vertical accelerations enable an extended regime to be exposed to the reshuffling of fins’ boundary layer, thus generating highly positive thermal dissipation flow structures. Even though PCRG exhibits greater negative *S*(*a*_*x*_), it is deprived in the vital negative *S*(*a*_*y*_), consequently dampens the strength of flow circulation and forced convection capabilities. Nevertheless, the greater displays of such events as compared with SFG allow for *Nu*(PCRG) > *Nu*(SFG), even though the numeral polarity of *S* registered similarity. Conversely, NG and RG demonstrated moderate flow circulations, hence the low *Nu*. In general, the extreme decelerated flow events are capable of forming intense flow vortices, which is beneficial in disruption of fins’ boundary layer. Further research is still required to uncover the *S* and *K* profiles at different inter-fin locality, in order to uncover the overall flow structures that are preferable for maximising forced convection of plate-fin heat sink.

### Power spectra density (PSD)

**Figure 9:**
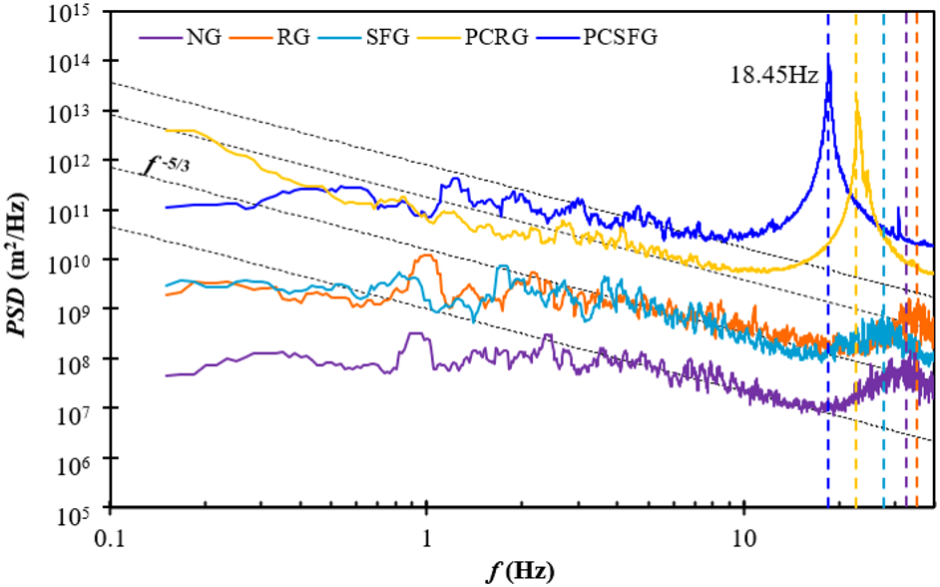
The 10-lapse period moving averaged PSD of velocity fluctuation induced with various grids at *Re*_*Dh*_ = 22.0 × 103.

When comparing the effects of various grids in their heat transfer performance, it is relevant to consider the PSD of flow, which deciphers the strength of velocity fluctuations in accordance with the frequency domain. Such feat is achieved by utilizing Fourier transform on the non-normalized autocovariance function of velocity fluctuation with respect to time, and is defined as follow:

11 $$P\left( f \right) = \mathop \smallint \limits_{ - \infty }^{\infty } r_{f} \left( t \right)e^{ - j2\pi ft} dt$$

12 $$r_{f} \left( t \right) = f^{^{\prime}} \left( t \right)f^{^{\prime}} \left( {t + \tau } \right)$$

where *P*(*f*) represents the PSD as a function of frequency *f*, *r*_*f*_ the non-normalized autocovariance function of velocity fluctuations and *j* the imaginary unit. The PSDs achieved by the 2D planar grids under influence of Re_*Dh*_ = 22 × 10^3^ are illustrated in Fig. 9. It is worthwhile to mention that the profile was subjected with 10-lapse period of moving average to minimize random noises.

Clearly, the energy level demonstrated by the partially-covered grids are much higher compared to the fully-covered counterparts, viz. an impressive increase of approximately × 10^2^, with NG recorded at the lowest energy state. The phenomenon coincides with the *I* profile in Fig. 5b, indicating a high coherence between the energy level and turbulence intensity, i.e. high *I* give rise to high PSD, and supported by findings in previous literatures^27,34^. It is noticed that the *P*(*f*) of all grid configurations decreases with increasing *f*, but proceed to surge at higher frequency domains, thereupon a second stage decay. Interestingly, the initial *P*(*f*) decay regions exhibit a reduction profile similar to that of Kolmogorov-law, i.e. with exponent approaching −5/3. Most grids are observed to follow the decay exponent over a wide range of frequencies except for PCSFG, where the *f*^−5/3^ is only documented at a narrow band, i.e. approximately between 4 Hz < *f* < 7 Hz. Such dissimilarity recorded for PCSFG, in particular the constricted range of *f*^*−5/3*^ regime, might indicate the formation of a distinct and unique flow structure. As seen in Fig. 9, the frequency ranges for PCSFG between 0 Hz < *f* < 4 Hz demonstrated a smaller decay, thus maintaining a higher energy level as compared to other grid configurations. As a result, energy carrying eddies of ample frequencies and length scales pierce through the inter-fin regions and support potent forced convective heat transfer. Elimination of the largest fractal bar at PCSFG mid-segmentation undermines wake formations and reduces hindrance to the streamwise flow structure. Eddies shed from various fractal bar thicknesses experiences lesser flow obstruction owing to minimal recirculation impacts, thus enabling coherent distribution of energy across vortices of all length scales. With the multitude energetic eddies shuffling the flow boundaries, force convection of heat along fins’ surfaces would be more efficient, thus realizing the superior *Nu* achieved by PCSFG.

As previously mentioned, the energy level surges at higher frequency ranges, and is exceptionally pronounced for the partially-covered planar grids. The vertically aligned mid-plane separation induces a distinct flow fluctuation, results in a powerful vortex shedding effect, which justifies the excitation at *P*(*f*) peak^17,35^. Moreover, the raised energy levels are observed to span over a wide range of frequencies, with range of PCSFG > PCRG. The phenomenon may imply that partially-covered grids are capable of generating broad array of high energy multilength scale eddies through (i) first stage grid-separation induced turbulence and (ii) second stage plate-fin eddies filtration for an intense vortex shedding process. As PCSFG comprised of different fractal bar thicknesses, there would be greater variations in eddies length scale, hence ampler variety of frequencies. Contrariwise, utilization of fully-covered grids masked the powerful vortex shedding effect, and is further subdued under NG configuration. The usage of SFG generates substantial turbulence intensity at centreline of *x/D*_*h*_ = 0.125, and one would infer that the energy level would be greater than RG, as *I*(SFG) > *I*(RG). However, the presence of largest grid bar on SFG produces sizeable wake with substantial flow recirculating in it. This causes the breaking of vortical structures which creates a less pronounce vortex shedding formation^28^. Even so, SFG prominent heat transfer, viz. *Nu*(SFG) > *Nu*(RG) is due to the effective distribution of flow kinetic energy in agitating fins’ boundary layer, as indicated by the wider spread of particle trajectory, especially in the spanwise direction. As for NG, the flow momentum is greatly sabotaged, causing the vortex shedding and energy level to subside. Surprisingly, the energy profile demonstrated by NG and RG are very much identical, revealing the impact of 2D planar grids in raising the preferable flow energy levels for forced convective heat transfer.

Upon closer inspections, it is noticed that the *P*(*f*) peaks are recorded at different frequencies, with the lowest realization of *f* = 18.45 Hz by PCSFG, thereupon PCRG, SFG, NG, and RG ranked in ascending order. The differences in frequencies might suggest the presence of an optimum fluctuation frequency *f*_*λ*_ that maximizes heat transfer through forced convection in plate-fin heat sink. As suggested in^30^, that velocity fluctuation frequencies at the two extremes of *f*_*λ*_ are ineffective in thermal transfer, as high frequencies (*f*/*f*_*λ*_ ≫ 1) contributed to diffusive effects, whereas low frequencies (*f*/*f*_*λ*_ ≪ 1) appeared as quasi-steady. Moreover, flow fluctuations at high frequency sub-range may be too rapid for any reshuffling of boundary layers to take effect. The particle’s trajectory projection illustrated in Fig. 6a, b supports the preceding statements, as high frequency fluctuations by PCRG and SFG appear to develop diffusive characteristics in the time-lapsed particle trajectory, causing the extensive contribution of turbulent kinetic energy confined around the shedding frequency to be ineffective in enhancing plate-fin heat transfer. As for PCSFG, the *f* is presumed to be approaching *f*_*λ*_, thus resulting in the formation of dense and intense annulus trajectory that directs the turbulent kinetic energy along the boundary layer for maximum agitation. In short, *f*_*λ*_* ≈* 18.45 Hz provides adequate velocity fluctuations for direct boundary layer restructuring, whist containing sufficient flow momentum to effectively enhance thermal dissipation.

The overall high vibrational energy demonstrated by partially-covered grids at centreline of *x/D*_*h*_ = 0.125, especially that of PCSFG offers high potential in localized heat transfer applications. It inherited certain features of SFG, whereby it is capable of generating multilength-scale eddies, whilst raising the energy level to × 10^2^ magnitude higher as evident in Fig. 9. Such results proves that the novel design of PCSFG has superior characteristics for thermal transfer as compared with SFG, thus opening the pathway for the further exploration on other partially-covered grid designs and their corresponding flow structures on heat transfer applications. Future works may be undertaken to explore the possibility of leveraging PCSFG turbulence generation strength by amalgamating such equivalent configuration within computers or data centre servers’ central processing unit (CPU) cooling systems, whereby potent electronics thermal dissipation is highly essential. PCSFG could be directly embedded as part of the cooling module, located either windward or leeward of heat sink fan (depending on either a suction or blow through mechanism, respectively) to provide indispensable turbulence characteristics that maximises forced convection, and, in the meantime, retaining the unit compactness. Another possible implementation is to incorporate the optimized-PCSFG(s) within the HVAC tunnel/system for energy harvesting purposes, hence as one of the energy sustainable solutions^28,36^.

## Conclusion

An in-house developed SPTV system was utilized to investigate the underlining grid-induced flow structures that reinforce positive thermal dissipation of plate-fin array, which was numerically optimized at *δ* = 5 mm and *l* = 10 mm under *Re*_*Dh*_ = 22.0 × 10^3^. The 2D *Nu* contour plots were able to reveal augmentations to heat transfer using partially-covered grids, where high *Nu* was seen at wider coverage range of (*δ*, *l*). Such feature infers the possibility of reducing fin quantity through realization of larger *δ* in heat exchanger unit, thus reducing manufacturing and material costs. Furthermore, the increases of 42.9% and 29.2% in *Nu* were observed by PCSFG and PCRG, respectively, as compared to control NG. The enhancements were believed to be stimulated from preferable flow of (i) high *U*/*U*_0_, (ii) intense *I*, (iii) strong (*v’*, *w’*), (iv) small *L*/*δ*, and (v) negative *S*(*a*_*x*_, *a*_*y*_) with (vi) low *K* for maximum thermal dissipation. The distinctive flow structures originates from the coaction between fractal bar thicknesses in generating a veracity of eddy sizes, and the vertical segmentation in reducing flow hindrance while restructuring wake-flow interactions to achieve heightened mass flow rate in penetrating inter-fin regions. These coupling flow dynamics also developed a unique formation of ‘Turbulent Annulus’ which offers a small degree of predictability on turbulence in the periodic annulus oscillation that effectively reshuffle flow boundary layers. Lastly, the PSDs induced by partially-covered grids were of × 10^2^ orders higher than fully-covered grids, with a prominent peak at high frequency sub-range that indicates strong vortex shedding effect. An optimal frequency of *f*_*λ*_ ≈ 18.45 Hz was determined by PCSFG, which was believed to provide adequate turbulent kinetic energy for direct boundary layer restructuring, while maintaining the necessary flow momentum to ensure superior force convective heat transfer of plate-fin heat sink. The findings revealed the potential of PCSFG induced turbulence in enhancing thermal dissipation, especially at the centreline region, which may deem useful in industries where targeted cooling is required.

## Supplementary Information

Supplementary Information.
