# Supplementary material for: Partially-covered fractal induced turbulence on fins thermal dissipation
Source: Sci Rep. 2022 May 12;12:7861. doi: 10.1038/s41598-022-11764-x (PMC9098450; doi:10.1038/s41598-022-11764-x)
Supplement: Supplementary file 1 — Supplementary Information. [file 41598_2022_11764_MOESM1_ESM.pdf]

Partially-covered fractal induced turbulence on fins thermal dissipation

Soon Hong Chew[1]*, Su Min Hoi[2], Manh-Vu Tran[1] and Ji Jinn Foo[1]*

[1]School of Engineering, Monash University Malaysia, 47500, Bandar Sunway, Malaysia

[2]Faculty of Engineering and Technology, Tunku Abdul Rahman University College, 53300 Kuala Lumpur, Wilayah Persekutuan Kuala Lumpur, Malaysia

*Corresponding author: soon.chew@monash.edu, Foo.Ji.Jinn@monash.edu

Supplementary Information

S1. Refractive error correction

The equations utilized in the module for refractive error corrections are observed as below:

$$\bar{R} = \sqrt{(x_d - x_c)^2 + (z_d - z_c)^2} \tag{S1}$$

$$\theta_1 = \tan^{-1}\left(\frac{\bar{R}}{y_D + y_w + y_l}\right) \tag{S2}$$

$$\theta_2 = \sin^{-1}\left(\frac{n_1 \sin\theta_1}{n_2}\right) \tag{S3}$$

$$R = y_D \tan\theta_1 + y_w \tan\theta_2 + y_l \tan\theta_1 \tag{S4}$$

where (x_c, z_c) represents the coordinates of optical centre, (x_d, z_d) the camera detected particle centroid, $\bar{R}$ the observed displacement from optical centre, y_D the detected horizontal particle-to-wall distance, y_w the acrylic wall thickness, y_l the horizontal lens-to-wall distance, (θ_1, θ_2) the Snell's law angles, (n_1, n_2) the refractive indices of (air, acrylic), and R the corrected displacement from optical centre. Intrinsically, coordinates corrected for refractive error (x_r, z_r) can be determined through rationalizing for similar triangles, and are computed as follow:

$$x_r = x_c + (x_d - x_c).\frac{R}{\bar{R}} \tag{S5}$$

$$z_r = z_c + (z_d - z_c).\frac{R}{\bar{R}} \tag{S6}$$

S2. Perspective error correction

The equations utilized in the module for perspective error corrections are observed as below:

$$x_a = \frac{y_D + y_w + y_l}{y_f + y_w + y_l}(x_r - x_c) + x_c \tag{S7}$$

$$z_a = \frac{y_D + y_w + y_l}{y_f + y_w + y_l}(z_r - z_c) + z_c \tag{S8}$$

where y_f represents the frame-to-wall distance, and (x_a, z_a) the calculated coordinate of the tracer particle.

S3. Coordinates detection iterative module

The equations utilized in the iterative module for coordinate detection are observed as below:

$$y_{a+1} = \frac{y_r - y_c}{z_l + z_w + z_f}\left(z_l + z_w + 0.5z_f - z_a\right) + y_c \tag{S9}$$

$$z_{a+1} = \frac{z_r - z_c}{y_l + y_w + y_f}\left(y_l + y_w + 0.5y_f - y_a\right) + z_c \tag{S10}$$

where y_a represents the calculated y coordinate of tracer particle.
